# High-altitude vertical wind profile estimation using multirotor vehicles

**DOI:** 10.3389/frobt.2023.1112889

**Published:** 2023-03-03

**Authors:** Alexander McConville, Thomas Richardson

**Affiliations:** Flight Lab, University of Bristol, Aerospace Engineering, Bristol, United Kingdom

**Keywords:** UAS, atmospheric sensing, vertical profiling, wind estimation, UAS operations

## Abstract

Capturing vertical profiles of the atmosphere and measuring wind conditions can be of significant value for weather forecasting and pollution monitoring however, collecting such data can be limited by current approaches using balloon-based radiosondes and expensive ground-based sensors. Multirotor vehicles can be significantly affected by the local wind conditions, and due to their under-actuated nature, the response to the flow is visible in the changes in the orientation. From these changes in orientation, wind speed and direction estimates can be determined, allowing accurate estimation with no additional sensors. In this work, we expand on and improve this method of wind speed and direction estimation and incorporate corrections for climbing flight to improve estimation during vertical profiling. These corrections were validated against sonic anemometer data before being used to gather vertical profiles of the wind conditions around Volcan De Fuego in Guatemala up to altitudes of 3000 m Above Ground Level (AGL). From the results of this work, we show we can improve the accuracy of multirotor wind estimation in vertical profiling through our improved model and some of the practical limitations of radiosondes that can be overcome through the use of UAS in this application.

## 1 Introduction

Vertical profile estimates of wind speeds and directions are vital for understanding the atmosphere, for weather forecasting, and for the prediction of pollution and atmospheric particulate concentrations. Some of the most extreme examples of atmospheric particulates are the plumes of ash that are ejected by volcanic eruptions. Prediction of ash distribution based on wind speed and direction can be used to minimize disruption to aviation and effects on human health, livestock, crops, and other infrastructure, as discussed by Poulidis et al. (2018). One way to improve wind and weather forecasting is to increase the frequency and spatial resolution of vertical atmospheric profiles.

Vertical wind profiling is currently carried out using radiosondes launched by weather balloons, which have been described as having enormous value by Flores et al. (2013). They offer the capability to gather data in largely inaccessible regions at a relatively low cost compared to many other methods. Weather balloons have been used to map changes and developments in the planetary boundary layer (PBL) throughout the day, such as in the study conducted by Zhang and Anthes (1982). These balloons carry sensing equipment to the upper atmosphere, where the balloons pop, letting the equipment to fall on the ground, on parachutes. These piles of debris are often unrecoverable and lead to pollution in the environment where they land (O’Shea et al., 2014). However, there are limitations when using radiosonde-based balloons for atmospheric sensing due to their lack of re-usability or the high levels of infrastructure required for tethered balloons; therefore, the frequency of launches is low, which can be problematic, as mentioned by Bhumralkar (1976), who parameterized the PBL, but it was concluded that far too little information was available on the PBL, leading to high levels of uncertainty in modeling. Dabberdt et al. (2004) looked at the increasing emphasis being placed on air quality forecasting and emergency response and reviewed the current state of the art in measurement systems by focusing on those used in the PBL, as this is the main consideration for the transport of pollutants and dispersion modeling; this would require a system for modeling other than radiosonde-based balloons throughout cities or other complex environments. To overcome these issues, we consider an approach using a UAS as an atmospheric sampling device. When developing vehicles for data collection, the type of flight regime that is desired must be considered. This was highlighted by Holland et al. (1992), alongside the World Meteorological Organization and the International Council of Scientific Unions in 1992, who considered the use of autonomous aircraft for their use in meteorology, focusing on stratospheric operations. A concept was developed for a <20 kg vehicle with on-board meteorological sensors to provide radiosonde-quality data. By 2001 (Holland et al., 2001), the Aerosonde had been developed into an autonomous meteorological data sampling instrument with the capability of operating for 2 days and covering more than 4000 km, putting this vehicle’s capability closer to the grade of manned aviation than many small-scale UASs. The vehicle was an early step toward the use of these small-scale UASs in meteorology. Other vehicles outside of conventional fixed wings have also been considered. Elfes et al. (1998) considered the use of airships for low-speed and low-altitude exploration and monitoring tasks and determined a number of benefits to them over conventional configurations under certain mission requirements. Chilson et al. (2019) considered the concept of routine small-scale UAS deployments to capture information on vertical profiles of the thermodynamics and the kinematic state of the atmosphere in conjunction with other weather observations to improve weather forecasting. Due to the simplicity of the systems and ease of development and operations, multirotor UASs have been widely used as atmospheric sensing systems. In this work, we focus on the capacity to produce vertical profiles of wind estimates, specifically those with a purpose well suited to the capabilities of rotorcraft.

In recent years, UASs have become an invaluable tool in many sensing applications due to their ability to carry out sensing over long ranges, in unsafe conditions, and in 3D space. Villa et al. (2016) surveyed the use of UASs in air pollution monitoring. It was found that pollution monitoring from ground-based sampling and, more recently, from aerial observations and satellites is limited when sources are moving or in complex environments. These problems can be overcome through the use of UASs as they can offer high-resolution sampling at varying positions and times, which is something not feasible through many of the conventional approaches to sensing. Schellenberg et al. (2019) and Schellenberg et al. (2020) used a fixed-wing aircraft to carry out volcanic plume sampling flights multiple times over an operating range of 9 km and at an altitude of above 4000 m, showing the capacity for these vehicles to be useful in data gathering at greater ranges and in circumstances that would be considered too dangerous for a manned aircraft. McGonigle et al. (2008) showed the capacity for UASs to be used for remote gas monitoring in hazardous conditions. Through the use of a UAS, CO_2_ fluxes were measured alongside measurements of SO_2_. Using a UAS for the aforementioned cases allows us to improve our understanding of conditions in hazardous environments and gather data that would be prohibitively risky and expensive to gather through any conventional methods. Another advantage of using a UAS for this kind of sensing is automation, as shown by Ma et al. (2004), who used a fixed-wing UAS to record measurements under autonomous flight conditions. The advantages of the platform included the light weight and high flexibility of the system, with significant ranges of up to 100 and 5 km altitude, making it a viable system for remote areas, although this would be limited by current regulations due to safety concerns more than any physical limitations of the vehicle.

Multirotor UASs have been considered previously for wind estimation, though this has been constrained to the hover condition, and many methods limit the approach to similar atmospheric regimes as where the modeling takes place. Palomaki et al. (2017) initially used multirotor vehicles as wind sensors by building a relationship between the angle of the body of the vehicle and the measured wind speed data from a sonic anemometer. A number of other approaches to modeling this relationship have been tested with varying degrees of success. Crowe et al. (2020) used more data-driven approaches, such as K-nearest neighbors and an LSTM, to build the relationship using similarly gathered data. Marino et al. (2015) evaluated multirotors as flying wind sensors for use around tall buildings and proposed another approach by mapping the power consumption of individual rotors to oncoming flow vectors. This approach was found to have a limited range in which it was accurate but did give greater insight into the aerodynamics of multirotor flight and the conditions under which the vortex ring state (VRS) develops for multirotor UASs. Wang et al. (2018) also described methods for calculating a rotor craft’s thrust and drag coefficients, which can be important in calculating the wind drag and airspeed. This work expands on some previously described methods by taking into account the acceleration of the vehicle. Varentsov et al. (2019) focused on the *in situ* observation of meteorological measurements in the atmospheric boundary layer. Using a mass market vehicle (DJI Phantom 4) with some additional sensing hardware, their work was able to show that even commercially available consumer vehicles have the capacity to be useful in atmospheric sampling by validating their readings against automatic weather stations.

To determine the value of using a UAS in meteorology, several studies have incorporated data gathered from UASs into meteorological forecasts. Sun et al. (2020) incorporated UAS sounding data from an RV Polarstern cruise in the Weddell Sea and evaluated their impact on the Polar Weather Research Forecasting model. This work was limited to the bottom 1 and 2 km of the atmosphere. Sun et al. (2020) found that the assimilation of UAS sounding data has a positive impact on accuracy, with a previous study finding that when significant amounts of radiosonde soundings were available, models improved significantly. The use of UASs also allows a greater degree of resolution and detail on changes in the environment allowing improvements to current modeling approaches through greater understanding, as described by Kral et al. (2018). By incorporating innovative sampling through UASs and conventional measurement approaches such as automatic weather stations, the structure of the atmospheric boundary layer (ABL) could be resolved at a high resolution.

For our vertical profiling consideration, we consider other factors, such as how the change in density of the atmosphere can change dramatically across a single profile and how the climbing velocity of the vehicle can change the system. In this paper, we improve on current wind estimation methods based on the vehicle response by expanding the capabilities to include climbing flight and by describing the assumptions and corrections made to account for vehicle motion when estimating wind and density variation in the atmosphere. We validate these assumptions by comparison of data measured using a sonic anemometer mounted on top of the vehicle and carrying out a variety of different climb rates for comparison. This was carried out at the Snowdonia Aerospace Center with extended permissions for flights up to 1000 ft.

Finally, we apply this sensing method to collecting vertical profiles in the region around Volcan de Fuego in Guatemala. Here, vertical profiles are carried out up to altitudes nearing 10000 ft AGL or over 13000 ft AMSL and compared with data captured using commercially available off-the-shelf balloon-launched radiosonde systems across a number of days and different times of the day. In addition to wind speed and direction data, data were captured on the temperature and humidity of the atmosphere as a further demonstration of the capabilities of these vehicles.

## 2 Bristol University Drone Sonde

For our purpose of high-altitude vertical wind profiling and to act as a testbed for the future of meteorological data sampling with a UAS, a custom vehicle was developed. The vehicle is built around a conventional open-source autopilot to ensure safe flight control is managed effectively but equipped with additional sensing and logging capabilities and the capacity for video recording or live video streaming for long-range operational awareness and data collection in Beyond Visual Line Of Sight (BVLOS). Our use case requires high levels of endurance and efficiency with the capability to fly across a range of atmospheric densities for vertical profiling and significant distances from the ground station. Due to the increased energy of a vehicle with a higher weight, an upper weight limit of 5 *kg* was chosen during the design process, with a lower weight being more desirable.

The Bristol University Drone Sonde (BUDS), as the platform is known, shown in Figure 1, is designed to work in the region of the ABL toward the altitudes of general aviation of more than 10,000 *ft*. The vehicle was to be used on operations in difficult environments and was, therefore, designed to be collapsible, lightweight, and easy to deploy. The vehicle characteristics are summarized in Table 1.

**Figure:**
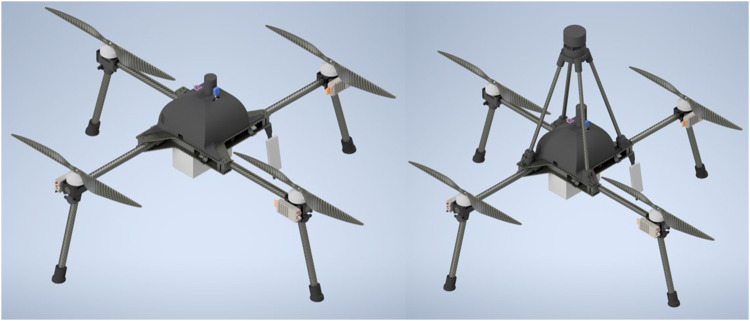
FIGURE Renders of the BUDS system with and without a sonic anemometer mounted.

**TABLE 1:** Description of the physical characteristics and hardware components of the BUDS systems.

Configuration	X
Motor-to-motor distance	0.9 m
Motors	AntiGravity 4006
Propellers	15x5″ Carbon fiber
Battery	6S 10,000mAh LiPo
Mass	4.0 kg
Autopilot	Pixhawk Cube Orange
Firmware	Arducopter V4.0.1
Additional computation	Teensy 4.1
Additional sensors	BME680 temperature and humidity
-	DHT11 temperature and humidity

Additionally, BUDS can include a mounted sonic anemometer. An FT742 2-axis sonic anemometer, with measurement characteristics of the anemometer, as shown in Table 2, was mounted sufficiently far away from the propellers such that the measured data are not affected by the flow. While this does increase the overall mass of the aircraft and reduce performance, it is a necessary addition to validate our wind estimation approach.

**TABLE 2:** FT-742 sonic anemometer measurement characteristics (FT-Technologies, 2021).

	Range	Resolution	Accuracy
Wind speed	0–75 m/s	0.1 m/s	±0.3 m/s (0–16 m/s)
Wind direction	0–360°	1°	4°

## 3 Wind estimation using multirotor vehicles

**FIGURE 2:**
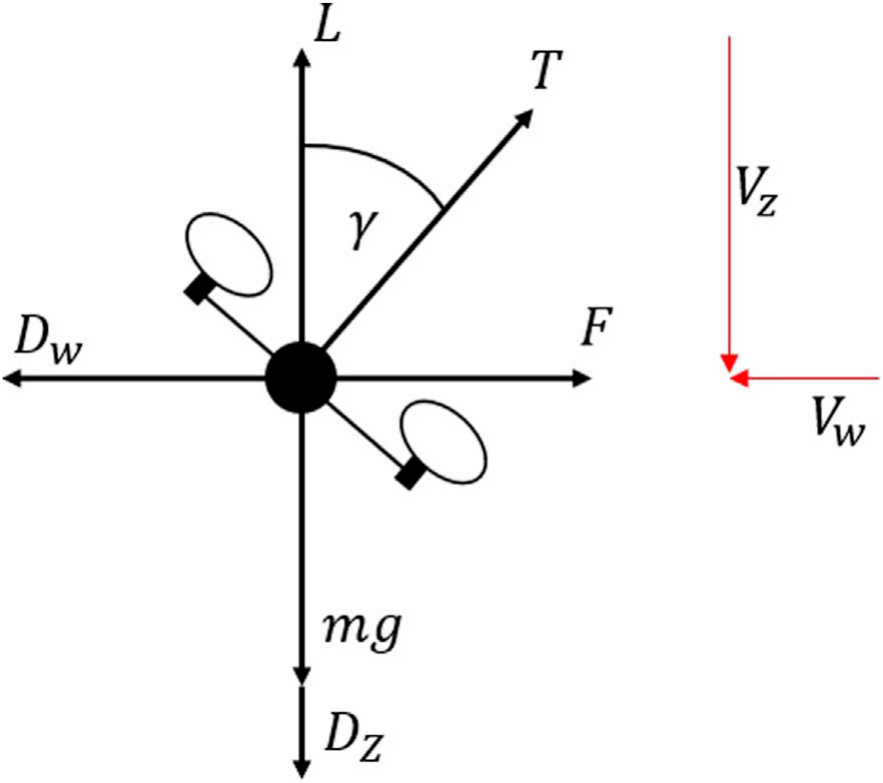
Free-body diagram of the UAS in climb.

The modeling approach used to estimate wind speed is described in detail in the study by McConville et al. (2022), where the vehicle is flown through a series of simple maneuvers to develop the wind estimation model under hover conditions. This model is then developed upon in this work to account for the motion of the vehicle while climbing and variation in atmospheric density. The estimation methods involving a multirotor UAS in most current works define a relationship between the body angle described in Eq. 2 and the wind speed acting on the vehicle using external wind speed measurement often from a sonic anemometer or similar sensor. The model developed for this vehicle, however, requires no additional sensors in the modeling process and relies only on vehicle maneuvers to build the estimation functions. These models are developed to estimate the wind under hover conditions based on a force balance, where the lift or vertical component of force is equal to the vehicle weight and the lateral component is equal to the force acting on the vehicle caused by the wind. By relating the angle required to generate this force, wind speed and direction can be determined while hovering, as shown in Figure 2.

Here, we discuss several steps taken to improve the model and correct for vehicle motion when estimating wind speed and then compare these values against those of the sonic anemometer mounted on the vehicle frame. The first step allows us to describe the force required to overcome particular wind speeds given particular body angles. Doing this allows us to easily account for changes in density caused by significant changes in altitude. If we define our model generated through maneuvers in this manner, we build a relationship between the force being generated during hover, where lift must equal the weight of the aircraft *mg*, and the airspeed being experienced, and by considering a multirotor in hover, we can define the system as described in Eqs 1–3, where *F* is the force being generated to resist the drag *D*_*w*_ being caused by the wind acting on the body, γ refers to the total body angle which is a function of pitch *θ* and roll *ϕ*, *A* refers to the area in the flow, and *V* is the airspeed with *ρ* being the atmospheric density.

$$F=D_{w},$$ (1)

$$\gamma =\arccos\left(\cos\left(\phi \right)\cos\left(\theta \right)\right),$$ (2)

$$mg\tan\left(\gamma \right)=\frac{1}{2}\rho V^{2}AC_{D}.$$ (3)

Developing on this, we can define a lumped drag coefficient and area term *C*_*DA*_(γ) which is a function of the body angle of the frame, with units of *m* ^2^, for use in calculating the lateral drag. The process for this is described in Eqs 4, 5. From data gathered in flight, we can define a function for *C*_*DA*_, meaning an accurate measure of area and drag coefficient is not required for our wind estimation, as we can convert from the forces being experienced to velocities.

$$AC_{D}=\tan\left(\gamma \right)\frac{mg}{\frac{1}{2}\rho V^{2}},$$ (4)

$$AC_{D}=C_{DA\left(\gamma \right)}.$$ (5)

The modeling flight allows us to find a relationship between body angle, forces, and velocity, from which we can determine the lumped *C*_*DA*(γ)_ relationship previously described. It should be noted that to expand the range of body angles experienced, measured data from an alternate flight with the sonic anemometer was used to supplement the data used in the model.

**FIGURE 3:**
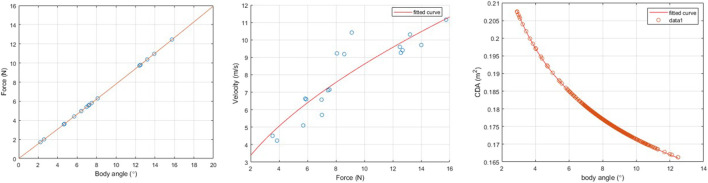
Summary of relationships captured during modeling flight.

Initially, we estimate the lateral force being generated by the vehicle across a range of body angles. This is shown in the first plot in Figure 3 and is a function of the mass of the aircraft as previously described as the aircraft maintains its altitude during translation or hover. This force and body angle relationship is then used to determine the overall force being produced by the vehicle laterally and the corresponding velocities that are being produced. This is shown in the second plot of Figure 3 and is in line with what would be expected, showing that as force increases, so does velocity with diminishing returns as drag increases with the square of velocity.

From this force–velocity relationship, it is possible to determine the previously described lumped drag term *C*_*DA*(γ)_. This is determined across a range of body angles and is shown in the final plot of Figure 3. Once a function for *C*_*DA*γ_ is determined, it is possible to calculate the airspeed based on an approximation of the force being generated laterally by the vehicle.

### 3.1 Climbing flight

As the climb rate increases, the assumption that the lift is equal to the weight of the aircraft no longer holds true. During a steady-state climbing flight, an additional force is required to maintain an ascent rate and overcome the vertical drag acting on the vehicle. At very low speeds, the additional thrust required to be overcome will have a minimal impact on the lateral force being generated. However, as vertical speeds increase, the increase in the thrust being generated will begin to have a noticeable impact on the force being produced, and therefore, the component of force required to overcome a given wind speed is achieved at smaller body angles. When climbing, the force acting vertically on the vehicle can be described as stated in Eq. 6 and shown in Figure 2, where *V*_*z*_ is the effective airspeed in the vertical direction caused by the climbing velocity and *V*_*w*_ is the lateral wind speed.

$$L=W+D_{z},$$ (6)

$$D_{z}=\frac{1}{2}C_{Df}\rho V_{z}^{2}\left(A_{\gamma }\right),$$ (7)

$$A_{\gamma }=A_{\min}+\left(A_{\max}-A_{\min}\right)\cos\left(\gamma \right).$$ (8)

With this increase in airspeed, the wind estimation method we described is in terms of the force required to overcome wind speed and includes the effects of the increased thrust required to produce a more accurate result under these circumstances. It is possible to determine the total force required to resist a given lateral airspeed in the hover. By assuming the vehicle is a flat plate, we approximate the force required to oppose the drag force and maintain an additional upward velocity. The coefficient of drag of a flat plate *C*_*Df*_ is considered to be 1.28, as stated by NASA (2022). The area in this case is approximated based on measurements of the plan-form areas both from above, *A*_max_ of 0.1027*m* ^2^, and one side *A*_min_ of 0.0603*m* ^2^, where it is assumed to vary between these two values in a sinusoidal fashion, as the area of the vehicle presented vertically varies with the body angle.

This increase in the vertical force required adjusts the relationship between lateral airspeed and velocity; it can also be described as a lateral force caused by the lateral air acting on the vehicle. The approximate additional thrust required is described as stated in Eq. 7. If attempting to sample over significant altitude ranges, higher climb rates may be required, which will increase the impact of this vertical drag component and the necessity for corrections alongside the corrections to more significant density changes.

With an improved estimate of the lift force determined by including the vertical drag component, we can describe *D*_*w*_, the force acting laterally on the climbing vehicle caused by wind, using Eqs 9–12. Here, our *C*_*DAγ*_ term, which we previously determined from Eq. 5, is used to describe the lumped coefficient of drag and frontal area being shown to the wind, which varies with the body angle γ.

$$F=L\tan\left(\gamma \right),$$ (9)

$$F=D_{w},$$ (10)

$$D_{w}=\frac{1}{2}C_{DA\left(\gamma \right)}\rho V_{w}^{2},$$ (11)

$$V_{w}=\sqrt{\frac{\left(W+D_{z}\right)\tan\left(\gamma \right)}{\frac{1}{2}\rho C_{DA\gamma }}}.$$ (12)

### 3.2 Anemometer corrections

As the anemometer is mounted on the vehicle and not directly correcting for motion, the measured data will be a combination of wind speed and the experienced motion of the vehicle. As the vehicle is tilted off center in flight and the anemometer system is rigidly mounted, it will only measure a component of the lateral airspeed. Therefore, the airspeed must be corrected initially, as stated in the following equation:

$$V_{\mathrm{asp}}=\frac{V_{\mathrm{measured}}}{\cos\left(\gamma \right)}.$$ (13)

The vehicle motion has an impact on the measured data, as changing the ground speed of the vehicle will change not only the measured velocity and direction of the flow but also the rotational rates of the vehicle as the sensor is mounted at a distance of 0.35 m above the center of gravity. To account for this, the data collected on board the vehicle are used to correct the measured data using the process described in Eqs 14, 15 by using the roll rate *p*, pitch rate *q*, yaw *ψ*, and distance *d*.

$$\begin{array}{ll} \left[\begin{array}{c} V_{Np}\\ V_{Ep} \end{array}\right] & =dp\left[\begin{array}{c} \cos\left(\psi \right)\\ \sin\left(\psi \right) \end{array}\right], \end{array}$$ (14)

$$\begin{array}{ll} \left[\begin{array}{c} V_{Nq}\\ V_{Eq} \end{array}\right] & =dq\left[\begin{array}{c} \sin\left(\psi \right)\\ \cos\left(\psi \right) \end{array}\right]. \end{array}$$ (15)

## 4 Sonic anemometer-based validation—Snowdonia Aerospace Center

To validate the corrections previously described in measuring the exact wind speed being experienced by the vehicle at any given moment, a sonic anemometer was mounted on the vehicle. The anemometer used was an FT technologies FT742 2-axis anemometer, shown in Figure 1, attached above the center of gravity of the previously described BUDS vehicle at a height of 0.345 m with the characteristics described in Table 2. The anemometer was mounted 0.345 m above the vehicle to remove the effect of the propellers on the sonic anemometer (Donnell et al., 2018).

The flights for these experiments took place at the Snowdonia Aerospace Center, where additional permissions from the CAA allowed us to reach an altitude of 300 m/984 ft instead of the conventional 120 m/400 ft limit allowed for UAS operations. A number of test flights were carried out by comparing the vertical profiles captured by using the sonic anemometer against a wind estimation determined from the vehicle response. Vertical climbs were carried out from 50 to 300 m AGL. This allowed a more significant sample window and range of wind speeds. Additionally, when attempting to reach higher climb rates, the higher ceiling allows more time once the desired velocity is reached.

### 4.1 Vertical profile comparison

**FIGURE 4:**
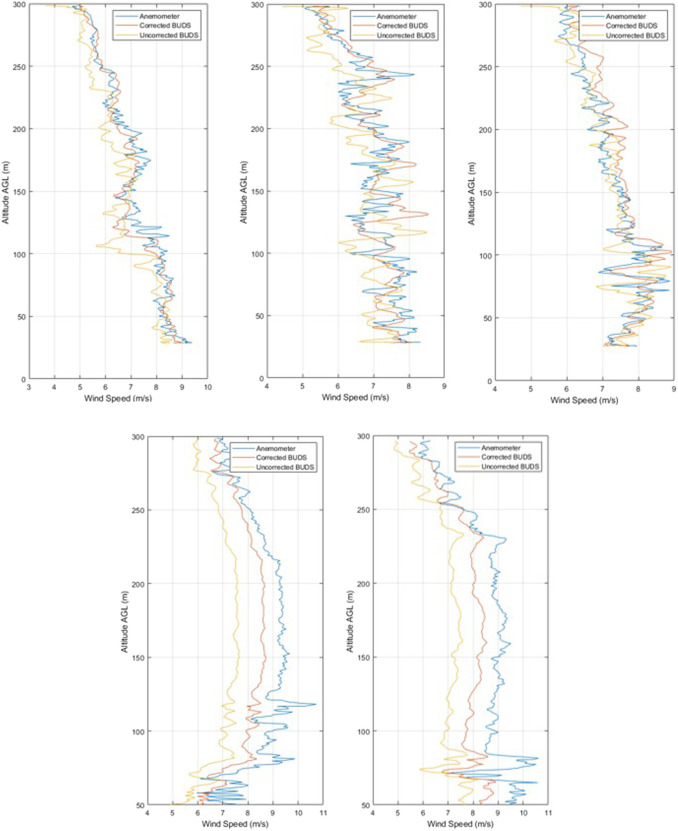
Comparison of measurements during climb at 4 m/s for ascents 1–5

After developing the wind estimation model, including non-hover condition corrections, and correcting the anemometer data for the vehicle motion, we obtain the vertical profiles shown in Figure 4.

To compare the accuracy of the results from the measured data and the estimation method, we compare the mean absolute error (MAE) described in Eq. 16, the root mean square error (RMSE) stated in Eq. 17, and the mean bias error (MBE) described in Eq. 18 between the estimated and measured wind speeds. In these equations, *y*_*i*_ describes the estimated values, *x*_*i*_ is the measured wind, and *n* is the total number of measurements.

$$\begin{array}{l} MAE=\frac{\Sigma_{i=1}^{n}|y_{i}-x_{i}|}{n}, \end{array}$$ (16)

$$\begin{array}{l} RMSE=\sqrt{\frac{\Sigma_{i=1}^{n}{\left(y_{i}-x_{i}\right)}^{2}}{n}}, \end{array}$$ (17)

$$\begin{array}{l} MBE=\frac{\Sigma_{i=1}^{n}|x_{i}-y_{i}|}{n}. \end{array}$$ (18)

Table 3 shows the results for vertical profiles accelerating from 0 m/s to a climb rate of 4 m/s and climbing from an altitude of 50–300 m AGL, compared with the corrected measurements of the sonic anemometer mounted above the vehicle. Ascents 1–3 are carried out in one flight, with ascents 4 and 5 being carried out in a second flight. In the data, we can generally see an improvement across the vertical profiles, with an improvement on the average of 0.58 m/s MAE.

**TABLE 3:** 4 m/s vertical profile results.

Ascent no.	Estimation	MAE (m/s)	RMSE (m/s)	MBE (m/s)
1	Corrected	0.51	0.68	−0.27
	Uncorrected	1.12	1.32	−1.06
2	Corrected	0.57	0.74	−0.03
	Uncorrected	0.98	1.21	−0.83
3	Corrected	0.46	0.64	−0.22
	Uncorrected	1.00	1.17	−0.95
4	Corrected	0.85	1.14	−0.61
	Uncorrected	1.53	1.80	−1.37
5	Corrected	0.90	1.18	−0.69
	Uncorrected	1.58	1.89	−1.44
Mean	Corrected	0.66	0.88	−0.36
	Uncorrected	1.24	1.48	−1.13

From the results, we can see that while in motion, the measured result varies slightly from the estimated values, but the MAE remains below 1 m/s for the corrected results in all of the profiles. This shows the effect the correction has while using the wind estimation model and the impact the thrust increase for climb has on the estimated values. As the density is very similar to that at which the model was developed, this effect is likely negligible; however, over a larger altitude range or higher altitude launch position, away from the region at which the model was developed, we may see a more significant impact from the density variation on the accuracy of our model.

When determining the effect of the various factors in our estimation equations, a simple sensitivity analysis was carried out on the 4 *m*/*s* ascents. Here, we compared the results of estimations where we changed a number of terms in the equation by ± 15%. The values we changed were the values of *C*_*Dz*_, the minimum and maximum values of the area *A*_min_ and *A*_max_, where both were reduced or increased by the same amount for each given test, and the measured body angle *γ*. The percentage change in error is summarized in Table 4. As is clear from Table 4, the major factor affecting the accuracy of our wind estimation under these conditions is *γ*, having a significantly larger effect on the percentage change in MAE. Interestingly, this effect also appeared to be non-linear, as gamma is present in a number of places throughout the equation and involved in trigonometric terms. Overall, the curve is mostly affected by the square root term in the equation. The effect that this will have on the error in the estimated reading will, therefore, change with the magnitude of the gamma. It is also clear that any error in the measurement of this angle will have a significant effect on the accuracy of the wind estimate; hence, this needs to be minimized through the selection of the sensors, the measurement and calibration process, and the post-flight data processing.

**TABLE 4:** Sensitivity analysis of factors in the wind estimation method.

	Absolute percentage change in MAE at ± 15%		
Ascent	*C*_*Dz*_ (+/−)	*A*_min / max_ (+/−)	*γ* (+/−)
1	0.82/0.86	0.82/0.86	7.88/88.30
2	0.07/0.11	0.07/0.11	33.70/42.96
3	0.65/0.72	0.65/0.72	24.00/98.72
4	1.21/1.22	1.21/1.22	31.29/68.97
5	0.90/0.91	0.90/0.91	35.85/63.51
Mean	0.73/0.76	0.73/0.76	26.97/72.49

#### 4.1.1 Climb velocity comparison

**FIGURE 5:**
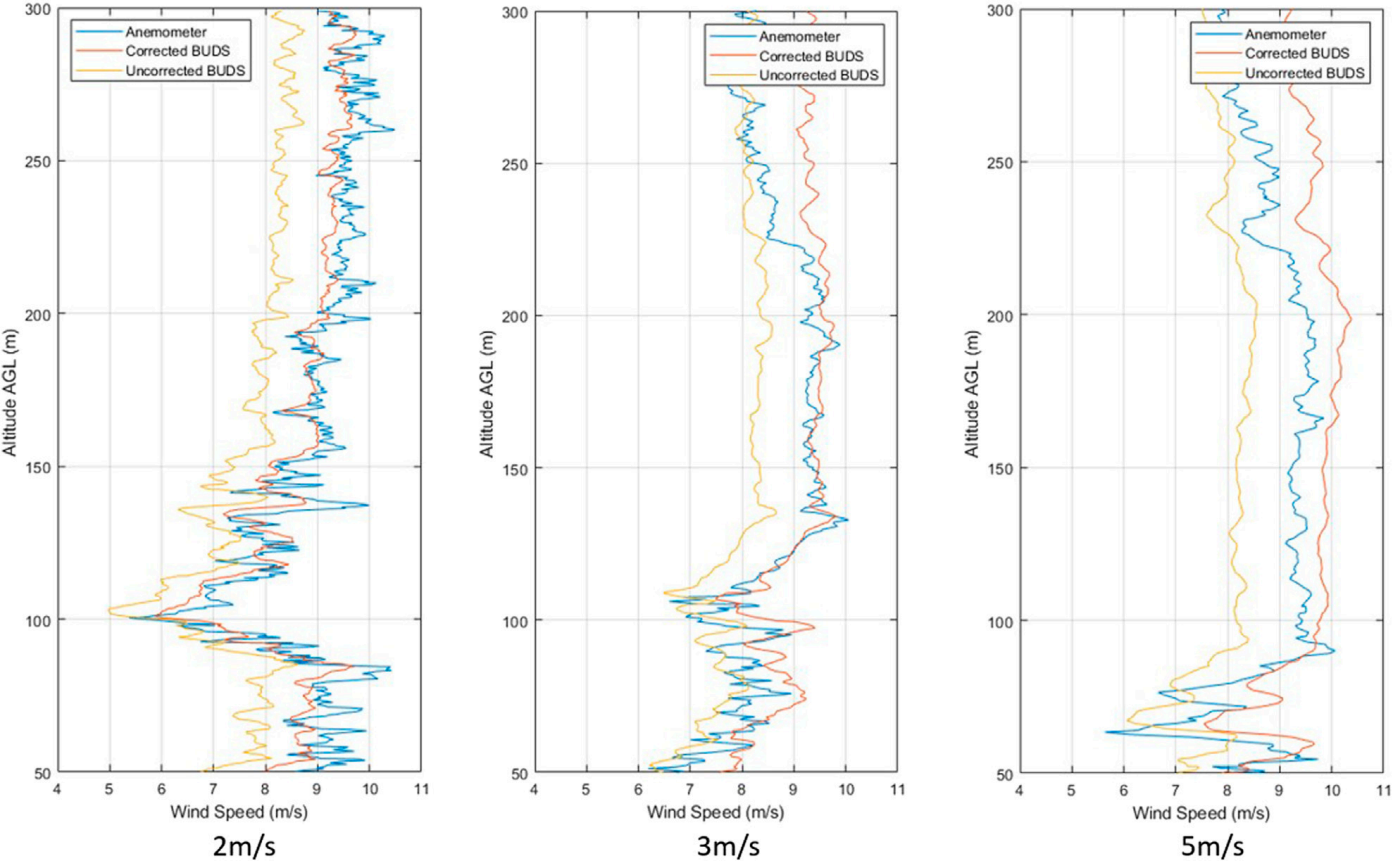
Climb measurement comparisons at 2, 3, and 5 m/s.

To determine if the rate of climb affects the accuracy or resolution of the measurements, a number of climb rates were completed. Climbs were carried out at 2, 3, 4, and 5 m/s, producing the profiles shown in Figure 5. Further ascent speeds were not possible with the vehicle in this configuration, as the vehicle was unable to maintain a 6 *m*/*s* climb with the additional 0.5 *kg* of mass beyond the expected design values for the vehicle.

By comparing the results of the measurement accuracy across a number of climb rates, we can see that the variation in error is below 0.25 *m*/*s*. Based on the results captured, we see a general trend of reducing accuracy with an increase in climb rate; however, all the measurements at 4 *m*/*s* seem to produce even lower error results than those at 2 *m*/*s*. This means that the variation in accuracy may well be caused by other factors in the wind, such as frequency of variation. Additionally, it is visible that the MBE tends to increase with the climb rate, which could be caused by an overestimation in the plan-form area or vertical drag estimation. Alternatively, the variation in MAE across the measurement range is within the 0.30 *m*/*s* uncertainty of measurements for the anemometer in this wind regime.

What we can see from these results is that the rate of climb does have the potential to impact the accuracy of the measured data but is also something that must be considered in the trade-off for the overall flight performance. Though further repeats would be useful to reinforce these results, based on current measurements, it appears that the effect of climb rate, across the range tested, has little impact on wind estimation accuracy.

By increasing the climb rate, it is possible to gather data over larger profiles or ranges as the vehicle is able to cover more distance with its given endurance; however, the resolution is reduced and there is uncertainty in the results. This is something to be considered in mission planning, where points or areas of interest may be traversed at lower rates than other sections of the flight with the aim to improve resolution in a particular region while still being able to reach a greater distance as may be required in hard-to-operate locations. Additionally, from the figures in Table 5, we can see that the climb rate of 4 m/s, which was used for further wind speed estimation flights, provides sufficient accuracy relative to the other options while maintaining high enough flight speeds to complete missions effectively.

**TABLE 5:** Accuracy at varying ascent rates (*mean of multiple results).

Ascent rate (m/s)	Estimation	MAE (m/s)	RMSE (m/s)	MBE (m/s)
2	Corrected	0.69	0.94	−0.25
	Uncorrected	1.31	1.53	−1.20
3	Corrected	0.76	1.01	0.51
	Uncorrected	0.89	1.11	−0.63
4*	Corrected	0.60	0.84	0.10
	Uncorrected	1.22	1.43	−1.12
5	Corrected	0.84	1.05	0.70
	Uncorrected	1.05	1.23	−0.91

The corrections made for the motion of the aircraft produce noticeable improvements in the vertical profiling sections. However, with the tested altitude range for the vertical profiles, the effect of density has not yet been fully determined.

## 5 Atmospheric profiling—Guatemala field campaigns

To compare the measurement capabilities in more realistic circumstances, the vehicles were taken to Volcan de Fuego in Guatemala. Two trips were carried out: the first, which took place in October 2021, was performed with the intention of initial hardware, systems, and process testing followed by the second test in January 2022, which focused on data capture. Guatemala was used as the testing environment for several reasons; previous experiments using UASs in that area provided good insight into difficulties faced, such as the import of vehicles, and knowledge regarding how to conduct operations with respect to the aviation authority the Direcion General de Aeronautica Civil (DGAC). Additional benefits of operating in this location are due to the highly active volcano. Airspace restrictions are in place; therefore, limited general aviation traffic takes place in the area. Due to the risks associated with living in this location, much of the area around the base and up the shallower regions of the slope of the volcano are uninhabited jungles, allowing for a high level of safety and higher-altitude operations in the region. The area does provide some difficulties in operating conditions with regards to access to components and batteries and very limited facilities. The topology of the local environment may also provide interesting features in the wind due to the ridges of the volcano and the prevailing wind direction.

The initial flights that took place in during the October 2021 trip proved the capability of the vehicle for reaching the desired altitudes. We were able to determine the upper limits of endurance under warmer conditions and higher altitudes, the best operating procedure regarding drone launch and descent, and balloon-sonde release methods. During the second trip, we took the understanding gained during our first trip and applied them to allow more effective data capture. The Windsond system is used as our conventional sounding approach, with its measurement characteristics detailed in Table 6.

**TABLE 6:** Windsond measurement characteristics (Windsond, 2019).

	Resolution	Response time	Uncertainty	Measurement range
Humidity	0.05%RH	6s	2%	0–100%RH
Temperature	0.01°C	6s	0.3°C	−40°C–80°C
Wind speed	0.1 m/s	-	5%	0–150 m/s
Wind direction	0.1°	-	Satellite dependent	0°–360°

### 5.1 Operational area and mission profile

The operational area for this work was based on the Instituto Nacional de Sismología, Vulcanología, Meteorología e Hidrología (INSIVUHMEH) Observatory, at the base of Volcan de Fuego. The airspace around the volcano was designated to us to carry out a variety of operations, and additional NOTAMs were issued to warn about UAS operations in the area.

**FIGURE 6:**
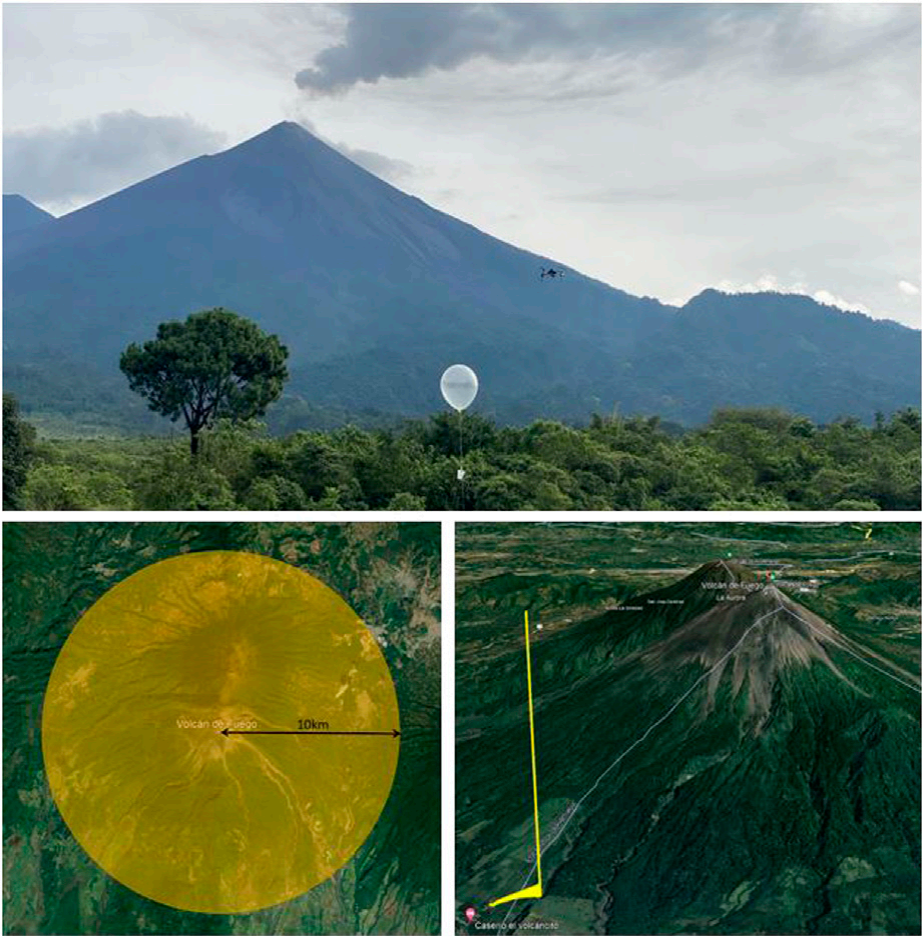
Imagery of the BUDS and Windsond system as well as the airspace available and mission profile taken during operations in Guatemala.

The airspace cordon was from ground level (1136*m*/3727 *ft* AMsL) up to a maximum altitude of 18, 000 *ft* AMSL with a cylinder centered on the summit of Fuego, with a radius of 10 *km*, as shown in Figure 6, with the flight profile taken.

The profiling mission is completed in the AUTO flight mode, climbing to 10 m above the take-off point. Here, the vehicle hovers for 15 s before rotating 90° and hovering again. From here, the vehicle moves away from any structure and over the vegetation while climbing to 20 *m* before moving 500 *m* toward the barranca (deep valley) while climbing to 100 *m* altitude. The vehicle then proceeds to climb at a constant rate up to the desired altitude. The vertical profiles were taken over the barranca to improve the safety of operations. Though unlikely, there is potential for someone to be beneath the vehicle when hovering in other regions , there are many small paths connecting fields used for agriculture, whereas the barranca will be completely uninhabited. Initially, the missions began with lower altitude climbs up to 500 m to thoroughly test the systems at ranges and altitudes not possible in the UK and determine the effect of lower density air on current draw and temperature on vehicle battery life. Following this, as confidence in the system was built up, the maximum altitude achieved was 4236 *m*/13898 *ft* ASML or 3000 *m*/9842 *ft* AGL.

### 5.2 Atmospheric profiles

The wind estimation model was redeveloped for the BUDS system without the mounted anemometer, and a number of profiles were captured in the Guatemala campaign with the vertical profiles displayed in this study. The corrected model is used to produce an estimate from the data gathered by the vehicle during these profiles.

**FIGURE 7:**
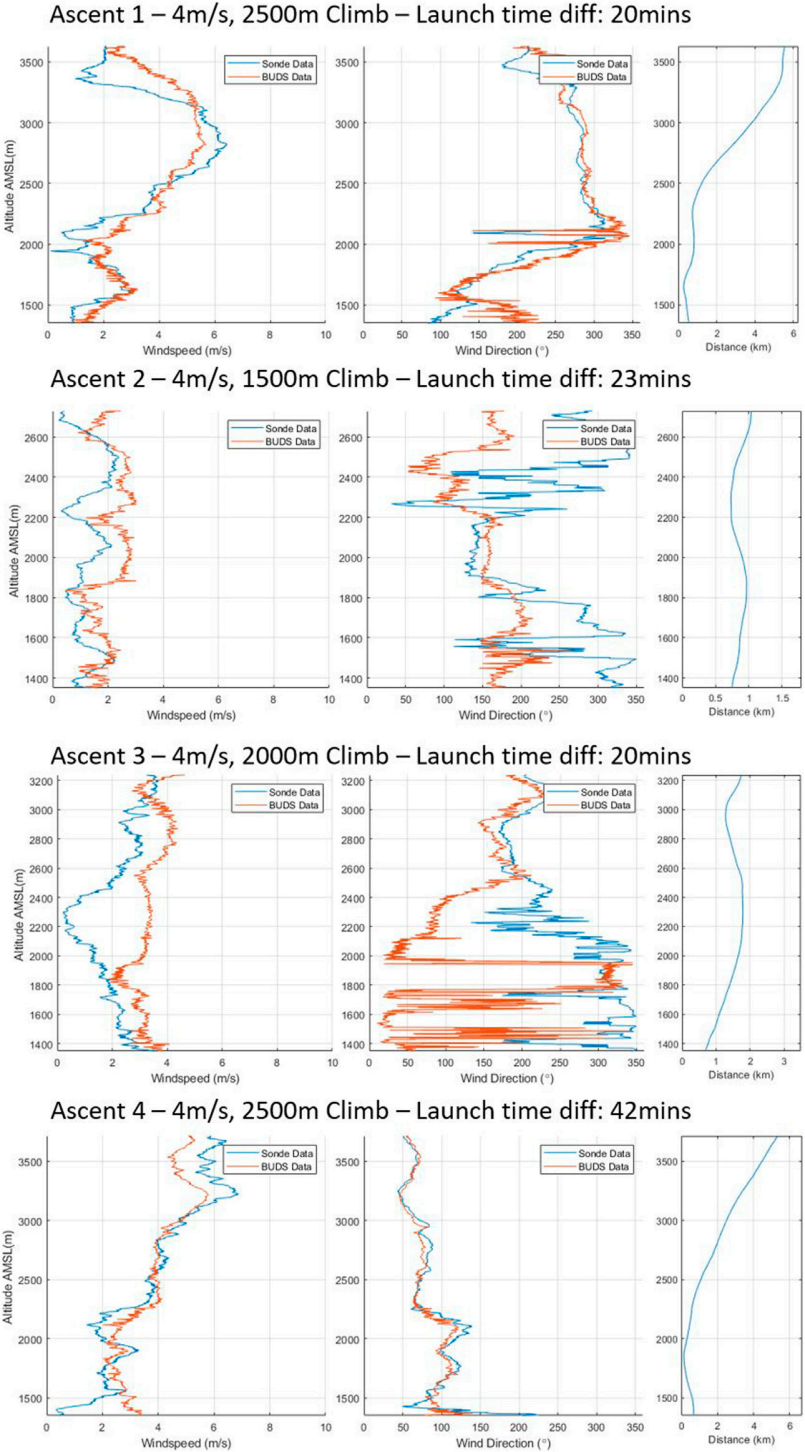
Ascents 1–4 of vertical wind profiles.

In ascent 1, in Figure 7 showing ascents 1 to 4, we see the wind speed measured by the vehicle and the sonde is very similar. There is a mean absolute error in the wind speed of 0.94 *m*/*s* across the entire ascent, whereas the wind direction error is 37.78°. A point of interest to note is that there is a significant deviation between the measured values in the direction when the wind speed is particularly low (<2m/s) , such as at the beginning of the ascent and throughout many of the other ascents. Toward the end of ascent 1, where the distance between measurement locations increased more significantly, we can see a further deviation in the measured wind speed and direction which is to be expected.

It is clear in ascent 2 that while the variation between the Windsond measured data and BUDS data for wind speed is fairly consistent, the variation in wind direction is much more significant. The low-wind-speed conditions particularly seem to impact the measurements of directions, with the difference between measurements reaching almost 180° in some places, with the Windsond system having significantly larger variation than the BUDS vehicle. This is an interesting result and brings into question the accuracy of one or both of the measurement approaches at low speeds, as can be seen in the third plot, showing the variation in the distance between the measurement positions. When the distance between measurement locations is compared to some of the other vertical profiles, the distance between the two points is relatively small, but the error remains high. Again, in ascent 3, while there is relatively low wind speed, the error remains high in the lower regions of the climb.

**FIGURE 8:**
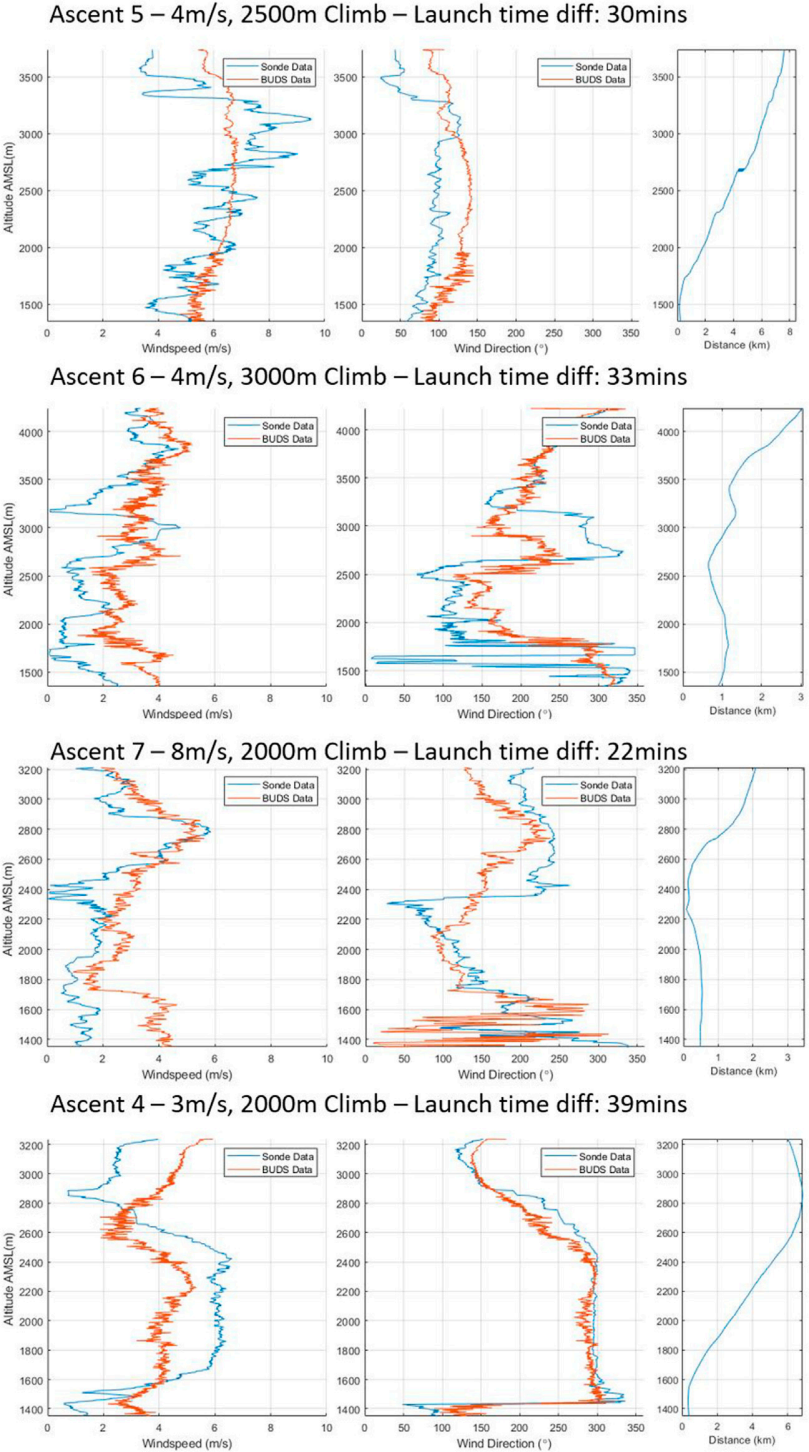
Ascents 5–8 of vertical wind profiles.

Ascent 4 is the best example of matching data profiles. Here, both speed and direction are almost identical. The mean absolute error for this complete profile is 0.79 *m*/*s* and 17.23°, showing that even in consistent conditions, the variation, particularly in the measured direction, can still be noticeable. Figure 8 shows ascents 5 to 8, with ascent 5 showing an interesting result where the conditions follow a similar average, but there is a significant variation in wind speeds being measured during the ascent by the sonde. In this particular ascent, the sonde was released second and the cloud base dropped significantly, which is visible in the wind speeds being experienced. In ascent 6, we see fairly consistent trends in the data being captured between measurement methods.

In ascent 7, we see a significant variation in the wind direction over the lower altitude in both the BUDS and the sonde measurement. There is also a large difference in wind speed in the lower 300 m of the ascent between the two measurement systems. This is one of the better examples of how turbulence may be affecting the systems differently based on the location or measurement tool. Finally, in ascent 8, we see that while the speed measurement has a consistent error, the direction estimation is very consistent between the two measurements.

The data gathered during the flights show a strong correlation between the vertical climb and data gathered from the radiosonde. Though the maximum wind speeds experienced in the profiles rarely exceed 8 m/s, we expect a high signal-to-noise ratio in the gathered data. The accuracy of the data gathered is summarized in 7. The errors in these results are more significant than those previously measured in the hover condition or in flight measurements from the sonic anemometer. Though the source of the errors is difficult to determine, as some are introduced by inaccuracies in the model, it is likely a significant portion of the variation in the measured wind speed and direction between the two measurements occurs due to the time and distance between when and where the measurements were taken. Additionally, to help minimize the error introduced by the major factor determined in the sensitivity analysis, *γ*, the body angle of the vehicle was re-calibrated for each ascent. Overall, the accuracy in speed estimation of the system with calibration based on the model developed through only maneuvers is shown to be consistent when compared with data gathered through conventional state-of-the-art methods, giving a mean speed error of 1.31 *m*/*s* across the measured profiles. Though the mean direction error of 49.05° is significant, the error is noticeably larger than the error measured during hover; therefore, it can be assumed to be caused by variation in the experienced wind. From Table 7, we can see that ascents 1, 2, and 4 all have a mean speed error below 1 *m*/*s*; however, interestingly, the direction errors across these ascents all have a variation from 17.23° to 119.25°, showing how an error in one does not coincide with the other. Additionally, there appears no correlation between the distance difference and time difference with the accuracy of the results, although there is likely an impact, and the conditions of the day and how they vary over time have the most significant impact on the variation between measurements.

**TABLE 7:** Summary of variation in ascent profiles.

Profile	Measurement	MAE	RMSE	MBE
Ascent 1	Wind speed (m/s)	0.94	1.17	0.37
	Wind direction (°)	37.78	57.04	16.89
Ascent 2	Wind speed (m/s)	0.86	1.10	0.70
	Wind direction (°)	101.22	115.20	−41.88
Ascent 3	Wind speed (m/s)	1.36	1.62	1.27
	Wind direction (°)	50.95	68.22	4.93
Ascent 4	Wind speed (m/s)	0.79	1.07	0.19
	Wind direction (°)	17.23	32.87	−8.53
Ascent 5	Wind speed (m/s)	1.10	1.34	0.41
	Wind direction (°)	30.9	35.02	29.42
Ascent 6	Wind speed (m/s)	1.48	1.76	1.36
	Wind direction (°)	56.48	73.09	8.21
Ascent 7	Wind speed (m/s)	1.87	2.19	1.41
	Wind direction (°)	57.05	68.74	−11.03
Ascent 8	Wind speed (m/s)	2.07	2.32	−0.64
	Wind direction (°)	41.16	60.90	−34.49
Mean	Wind speed (m/s)	1.31	1.57	0.63
	Wind direction (°)	49.05	63.88	−4.56

The results of this profiling approach show that this method can be used in place of or alongside conventional balloon-based radiosondes. Additional benefits come in the form of re-usability, regularity of sounding flights, and capacity for sampling across multiple locations easily, as the sampled location was 500 *m* away from the launch location. Additionally, sampling can be carried out at a reduced cost. The total build cost for the vehicle was approximately £2000 for a reusable system, with the vehicle still being able to continue to operate, while the radiosonde system costs totaled several thousand, and reuse was limited.

## 6 Conclusion

From the data gathered during the operations in Guatemala, we can see that wind profiles vary not only with time but also over relatively small distances. Vertical profiles can be carried out up to significant altitudes using UASs. There are several benefits to using these systems for this over the current balloon-based approach. These benefits include the ability to repeatedly collect data in precisely the same location, limit the use of disposable balloons for soundings, and have control over the system once it is airborne.

Some limitations have been highlighted in the use of multirotor vehicles for this purpose, with the major limit currently being the achievable altitude. Our vehicle reached altitudes of 3000 *m* AGL or above 4000 *m* AMSL; this range is useful for meteorology as an addition to the current radiosonde network, but to fully replace them, higher altitudes need to be reached. These altitude limits are imposed by battery and airframe constraints. By optimizing the airframe for a wider range of altitudes and minimizing weight, we may be able to improve the range. The other primary limitation is regulatory guidelines, which can vary by country. Without improving safety and confidence in these systems, it is unlikely that they will gain broad acceptance and use in this context.

## Data Availability

The raw data supporting the conclusions of this article will be made available by the authors, without undue reservation.
